# *EDA* Missense Variant in a Cat with X-Linked Hypohidrotic Ectodermal Dysplasia

**DOI:** 10.3390/genes15070854

**Published:** 2024-06-28

**Authors:** Stefan J. Rietmann, Noëlle Cochet-Faivre, Helene Dropsy, Vidhya Jagannathan, Lucie Chevallier, Tosso Leeb

**Affiliations:** 1 Institute of Genetics, Vetsuisse Faculty, University of Bern, 3001 Bern, Switzerland; stefan.rietmann@unibe.ch (S.J.R.); vidhya.jagannathan@unibe.ch (V.J.); 2 Dermfocus, University of Bern, 3001 Bern, Switzerland; 3 Unité de Dermatologie, CHUV-Animaux de Compagnie, Ecole Nationale Vétérinaire d’Alfort, 94700 Maisons-Alfort, France; noelle.cochet-faivre@vet-alfort.fr (N.C.-F.); hdropsy@gmail.com (H.D.); 4 BIPAR, Laboratoire de Santé Animale, INRAE, Ecole Nationale Vétérinaire d’Alfort, 94700 Maisons-Alfort, France; 5 U955-IMRB, Team 10-Biology of the Neuromuscular System, INSERM, UPEC, Ecole Nationale Vétérinaire d’Alfort, 94700 Maisons-Alfort, France; lucie.chevallier@vet-alfort.fr

**Keywords:** *Felis catus*, WGS, dermatology, skin, development, X-linked, precision medicine, animal model

## Abstract

Hypohidrotic ectodermal dysplasia is a developmental defect characterized by sparse or absent hair, missing or malformed teeth and defects in eccrine glands. Loss-of-function variants in the X-chromosomal *EDA* gene have been reported to cause hypohidrotic ectodermal dysplasia in humans, mice, dogs and cattle. We investigated a male cat exhibiting diffuse truncal alopecia with a completely absent undercoat. The cat lacked several teeth, and the remaining teeth had an abnormal conical shape. Whole-genome sequencing revealed a hemizygous missense variant in the *EDA* gene, XM_011291781.3:c.1042G>A or XP_011290083.1:p.(Ala348Thr). The predicted amino acid exchange is located in the C-terminal TNF signaling domain of the encoded ectodysplasin. The corresponding missense variant in the human *EDA* gene, p.Ala349Thr, has been reported as a recurring pathogenic variant in several human patients with X-linked hypohidrotic ectodermal dysplasia. The identified feline variant therefore represents the likely cause of the hypohidrotic ectodermal dysplasia in the investigated cat, and the genetic investigation confirmed the suspected clinical diagnosis. This is the first report of an *EDA*-related hypohidrotic ectodermal dysplasia in cats.

## 1. Introduction

One of the first scientific descriptions of hypohidrotic ectodermal dysplasia was provided in 1875 by Charles Darwin, who studied reports on a four-generation Hindu family from India with ten affected male relatives. These studies enabled him to deduce fundamental principles of X-linked recessive inheritance [1].

The clinical phenotype in humans involves sparse or absent hair, abnormal dentition characterized by partially missing teeth and remaining teeth exhibiting a distinctive pointed morphology, as well as a deficiency in various glands, notably sweat glands, resulting in heat intolerance [2]. This phenotype has been interchangeably termed hypohidrotic ectodermal dysplasia (HED), anhidrotic ectodermal dysplasia, or Christ–Siemens–Touraine syndrome [3]. The vast majority of human HED patients carry loss-of-function variants in the X-chromosomal *EDA* gene encoding ectodysplasin A [2,4]. Ectodysplasin A is a homotrimeric type II transmembrane protein with an intracellular N-terminus, a single transmembrane domain, an extracellular short collagen-like domain that mediates triple helix formation and trimerization and a C-terminal signaling domain that has sequence homology to tumor necrosis factor (TNF) [5,6,7]. Alternative splicing gives rise to two alternative transcripts from the ~400 kb *EDA* gene, which encode two protein isoforms termed EDA-A1 and EDA-A2 that differ by the presence or absence of two amino acids in the TNF signaling domain and bind to two different receptors [8]. The physiological functions of the shorter EDA-A2 isoform and its receptor are largely unknown. Expression of the longer EDA-A1 isoform during fetal development prompts the formation of many different ectodermal appendages, such as hair follicles, tooth buds or sweat glands. The signaling cascade involves extracellular proteolytic cleavage of the membrane-bound EDA-A1 by furin proteases to release a paracrine trimeric signaling molecule. The released soluble fragment can bind with its TNF signaling domain to the ectodysplasin A receptor (EDAR) on target cells. Activated EDAR recruits an intracellular adaptor protein termed EDAR associated via death domain (EDARADD), and the complex activates NFκB signaling to modulate the expression of target genes [9].

Loss-of-function of *EDA*, *EDAR* or *EDARADD* leads to identical clinical phenotypes in human patients [10,11]. However, the vast majority of human patients are due to *EDA* variants, and this specific form of the condition is termed X-linked hypohidrotic ectodermal dysplasia (XHED; OMIM #305100). The Leiden Open Variation Database currently lists 164 pathogenic or likely pathogenic variants [12]. Autosomal recessive or dominant inheritance is seen in very rare forms of HED due to variants in *EDAR* or *EDARADD*.

*EDA* variants causing hypohidrotic ectodermal dysplasia were also reported in mice [5,6], dogs [13,14,15,16] and cattle [17,18,19,20,21,22,23,24,25,26,27,28]. *EDA*-deficient dogs have been successfully used as animal models for therapeutic trials that are now ongoing in human patients [29,30].

This study was prompted by the presentation of a male cat with clinical signs resembling hypohidrotic ectodermal dysplasia in humans and other species. The aim of our study was to provide a detailed characterization of the clinical phenotype together with an investigation of the underlying causative genetic variant.

## 2. Materials and Methods

### 2.1. Ethics Statement

The affected and all 96 control cats in this study were privately owned. Blood samples were collected with the consent of the owners. The diagnostic work-up of the index case did not constitute an animal experiment in the legal sense. The collection of blood samples from control animals was approved by the Cantonal Committee for Animal Experiments (Canton of Bern; permit BE94/2022). All animal experiments were conducted in accordance with local laws and regulations.

### 2.2. Clinical Examination

The affected cat underwent regular general and dermatologic examinations. Regular monitoring of atypical dermatitis and chronic calicivirus was carried out for two years. Hematological and biochemical check-ups, urinary analyses and abdominal ultrasound scans were regularly carried out as part of the animal’s clinical follow-up.

The 96 control cats represented population controls without any reports of combined tooth and hair abnormalities. As the phenotype in the affected cat was very striking, we considered all 96 control cats as clinically unaffected.

### 2.3. DNA Isolation and Whole-Genome Sequencing

Genomic DNA was isolated from EDTA blood on a Maxwell RSC 16 or 48 instrument using the Maxwell RSC Whole Blood DNA Kit (Promega, Dübendorf, Switzerland). A PCR-free library with ~400 bp insert size was prepared from genomic DNA of the affected cat. The library was sequenced with 2 × 150 bp paired-end chemistry at 20× coverage on an Illumina NovaSeq 6000 instrument (Illumina, San Diego, CA, USA). The raw reads in fastq files were processed into a binary alignment map (bam-file) with respect to the F.catus_Fca126_mat1.0 genome reference assembly (GCF_018350175.1). Subsequently, single-nucleotide variants and small indels were called as described before [31]. The accession numbers of the sequence data were deposited in the European Nucleotide Archive and are listed in Table S1. Functional effects of the called variants were predicted with the SnpEff version 4.3t software [32] together with NCBI annotation release 105 for the F.catus_Fca126_mat1.0 genome reference assembly.

### 2.4. Variant Filtering

We filtered for private variants in the affected cat by comparing its genome sequence data to a control cohort comprising 96 publicly available WGS datasets from genetically diverse cats (Table S1). Variants in the affected cat that also occurred in at least one of the control cats were excluded from further analysis. In a second step, protein-changing variants were prioritized. We considered variants with an SnpEff predicted impact of “high” or “moderate” as protein-changing.

### 2.5. In Silico Pathogenicity Prediction

The online classification tools Polyphen-2 [33], PredictSNP [34] and MutPred2 [35] were utilized to predict the potential functional impact of the XP_011290083.1:p.Ala348Thr missense variant. The Mol*3D viewer [36] from the RCSB Protein Data Bank was used to visualize the protein structure 1RJ7 of the TNF signaling domain of the human ectodysplasin A protein [37].

## 3. Results

### 3.1. Clinical Phenotype

A 9-year-old neutered male European domestic shorthair cat was presented for pruritus of the limbs and abdomen. The cat had been adopted at the age of 7 years old from a rescue organization. The cat had been given regular external antiparasitic treatment.

**Figure 1:**
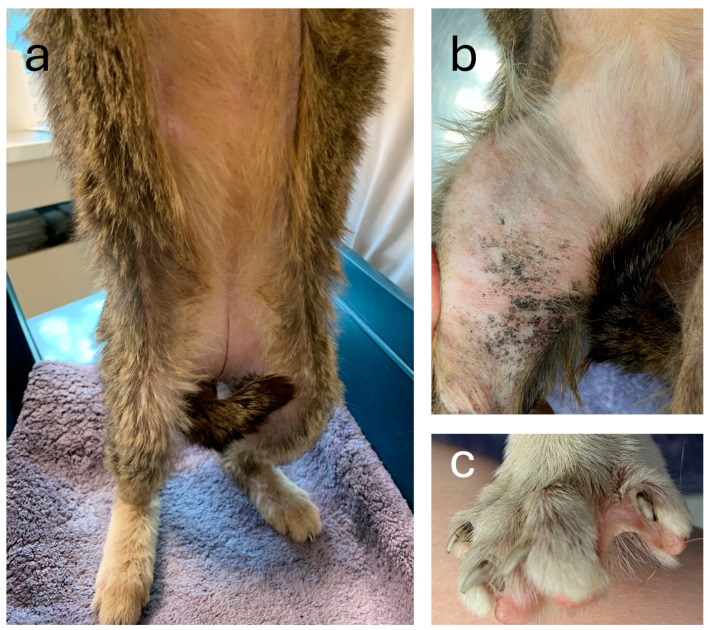
Diffuse hypotrichosis of the affected cat. (**a**) Complete abdominal alopecia and a lack of undercoat on the remaining haired skin. (**b**) Alopecia and hyperpigmentation of the inner thighs. (**c**) Alopecia of ungual ridges.

At presentation, diffuse truncal alopecia with complete alopecia of the abdomen, alopecia and hyperpigmentation of the inner thighs and alopecia of ungual ridges was evident (Figure 1). Coat inspection revealed reduction to absence of the undercoat with presence of guard hair only. Diffuse squamosis, follicular casts, diffuse erythema and overall loss of skin elasticity were also present. The hair loss had not changed since adoption, and the erythema and pruritus appeared a few weeks prior to presentation. The cat had a history of chronic rhinitis.

**Figure 2:**
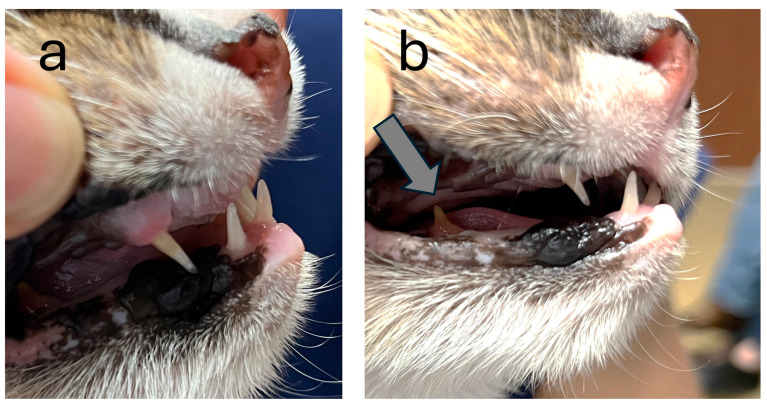
Abnormal dentition in the affected cat. Only the four canines and the two lower premolars with an abnormal, extremely pointed shape were present. The incisors, upper premolars and molars were missing. (**a**) Four canines are visible. (**b**) The right lower premolar is indicated with an arrow.

At the time of presentation, the cat had only six teeth, the four canines and two lower premolars. The incisors, upper premolars and molars were missing. The canines and the present premolars had an abnormal conical shape (Figure 2).

Blood hematology and biochemistry revealed only moderate eosinophilia and marked neutrophilic leukocytosis, probably secondary to chronic dermatopathy (Tables S2 and S3). Cushing syndrome was ruled out by abdominal ultrasonography, which revealed normal, normoechoic adrenal glands of normal size and shape, as well as normal blood and urine tests and a normal urine specific gravity of 1.050 (Table S4). FIV and FelV tests were negative. The owner declined skin biopsies for further histopathological examinations.

Altogether, the clinical signs of hypotrichosis, squamosis, follicular casts and dental agenesis, along with the exclusion of a metabolic disease such as Cushing syndrome, led to a suspicion of hypohidrotic ectodermal dysplasia [13,16,38,39].

### 3.2. Genetic Analysis

We sequenced the genome of the affected cat and compared the sequence data to 96 genetically diverse control genomes in a search for plausible causative variants. Several filtering steps were performed based on hypothetical dominant or recessive modes of inheritance, allele frequency in the control cohort and predicted variant impact. Variants in the known candidate genes *EDA*, *EDAR* and *EDARADD* were prioritized (Table 1 and Table S5).

**Figure 3:**
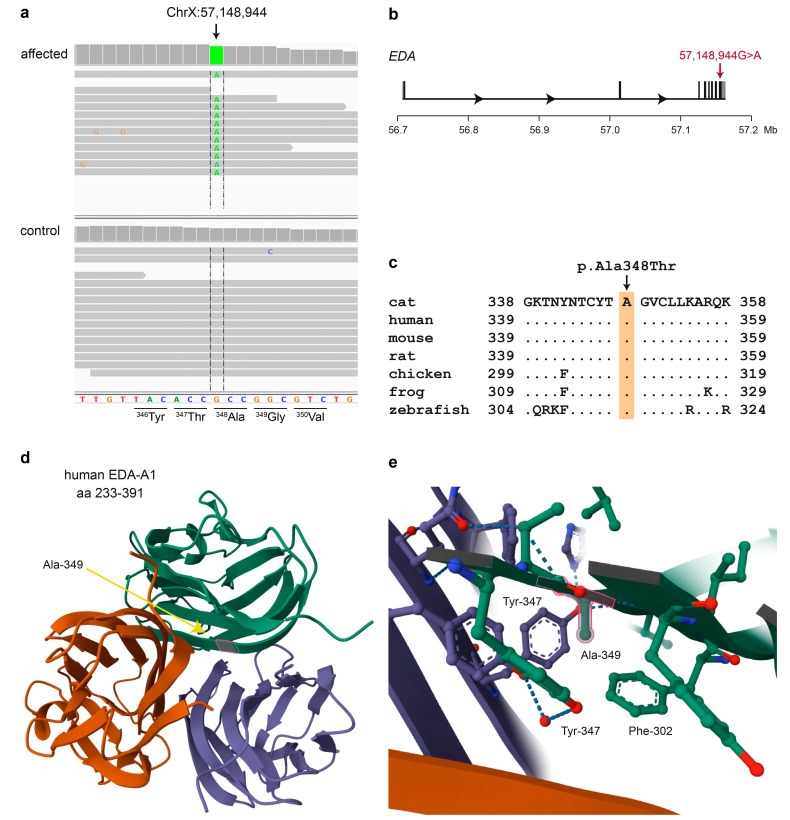
Details of the *EDA*:p.Ala348Thr variant. (**a**) Integrative Genomics Viewer screenshot of the short-read alignments from the affected cat and an unrelated control demonstrates the hemizygous c.1042G>A single-base substitution in the affected cat. The reading frame and amino acid translation of the reference sequence are indicated. (**b**) Genomic organization of the ~444 kb feline *EDA* gene with its 8 exons. The c.1042G>A missense variant is located in the last exon. (**c**) Evolutionary conservation of ectodysplasin A in the region of the predicted amino acid exchange. The alanine at position 348 of the feline ectodysplasin A protein is strictly conserved across all vertebrates. The sequences were derived from the following database accessions: cat XP_011290083.1, human NP_001390.1, mouse NP_034229.1, rat NP_001292172.1, chicken NP_001409628.1, frog XP_004916866.1 and zebrafish NP_001108537.1. (**d**) Structure of the human trimeric EDA-A1 TNF signaling domain [37]. Ala-349 (which corresponds to Ala-348 in the feline protein) is located within a β-sheet at the contact surface between the three monomers. (**e**) Details of the structure. The small methyl side chain of Ala-349 is densely packed between the phenyl-rings of Phe-302 and the phenyl-rings of two Tyr-347 residues from two different monomers, which form a hydrogen bond in the trimeric structure.

The analyses identified a single candidate variant on the X chromosome, NC_058386.1:g.57,148,944G>A. It is a missense variant in the *EDA* gene, XM_011291781.3:c.1042G>A, predicted to change an alanine into a threonine residue on the protein level, XP_011290083.1:p.(Ala348Thr). This predicted amino acid substitution is located in the TNF signaling domain of ectodysplasin A (Figure 3). The mutant allele was absent from 404 cat genomes of the 99 Lives Consortium [40,41].

In silico pathogenicity prediction tools classified the p.Ala348Thr variant as potentially deleterious. MutPred2 gave a score of 0.541, marginally above the pathogenicity threshold of 0.5. PredictSNP classified the p.Ala348Thr variant as deleterious with 72% probability. Polyphen-2 classified the variant as probably damaging with a score of 0.995.

The amino acid sequence of the TNF signaling domain is highly conserved between vertebrates. The alanine corresponding to the feline Ala-348 residue is invariable in all vertebrate sequences analyzed (Figure 3c). This alanine is located in a β-sheet at the contact interface between the three EDA-A1 monomers (Figure 3d). The small methyl side chain is in close proximity to three phenyl rings from neighboring amino acids (Figure 3e).

## 4. Discussion

In this study, we investigated a male cat with a syndromic phenotype involving partially missing hair and missing or abnormally shaped teeth. This combination of clinical signs was classified as hypohidrotic ectodermal dysplasia. This phenotype is highly characteristic, and especially in male individuals, the X-chromosomal *EDA* is the primary functional candidate gene. Many pathogenic variants in *EDA* have been reported in human patients [4,7,12,42,43], dogs [13,14,15,16] and cattle [17,18,19,20,21,22,23,24,25,26,27,28]. The history of chronic rhinitis in the affected cat might have been caused by defects in respiratory mucous glands, which are a common feature of hypohidrotic ectodermal dysplasia in humans, cattle and dogs [13,44,45]. Keratoconjunctivitis sicca, another frequent phenotype in human X-linked hypohidrotic ectodermal dysplasia due to defects in lacrimal glands, was not observed in the investigated cat.

For practicing veterinarians, it is important to consider all organ systems that may be affected in XHED to provide an optimal management of potential future cases. Special attention should be paid to good management of respiratory infections and surveillance of eye irritations due to possible gland defects in XHED-affected animals.

The identified *EDA* missense variant in the affected cat, p.Ala348Thr, is predicted to affect a single amino acid within the TNF signaling domain, located at the contact interface between the three monomers. Interestingly, the homologous human variant, p.Ala349Thr, has been identified in at least two independent human XHED families [7,43]. The available knowledge on the homologous human variant provides very strong support for the pathogenicity of the feline p.Ala348Thr variant. It seems conceivable that replacement of the alanine with the slightly larger and more polar threonine might interfere with correct assembly of the trimeric TNF signaling domain. The hydroxy group of the mutant threonine might potentially disrupt a hydrogen bond that normally bridges two adjacent tyrosine side chains from two different subunits [37].

At the nucleotide level, the substitution affects a CpG dinucleotide. CpG dinucleotides represent known mutational hotspots due to the spontaneous deamination of 5-methylated or unmethylated cytosines, which results in CG → TG or CG → CA substitutions as in the cat described herein [46].

The ~400 kb *EDA* gene is one of the largest genes in the mammalian genome. Many different types of deleterious sequence variants have been identified that lead to a loss of function of ectodysplasin A and result in XHED. These include single-nucleotide variants representing missense variants such as in the cat described herein and several examples in humans [7,42,43] and cattle [27,28]. Other single-base substitutions represent nonsense variants [19] or were reported to disrupt splicing [13,18]. Additionally, small coding insertions and deletions, either in-frame [41] or frame-shifting [20], and large structural variants were reported in XHED-affected individuals [17,25]. Exon 2 of the *EDA* gene is flanked by very large introns on either side, which complicates the correct splicing of the primary transcript. Splicing aberrations in XHED-affected cattle have been reported in an animal with a partial LINE-1 insertion into intron 1 that resulted in “exonization” of the inserted sequence and a non-functional transcript [21]. Finally, in three XHED-affected dogs, skipping of exon 2 was observed, although the genomic sequence of exon 2 and its flanking splice sites were unaltered. The genomic variant causing this splice defect has not yet been identified [14].

## 5. Conclusions

We characterized the clinical phenotype of a male cat with X-linked hypohidrotic ectodermal dysplasia (XHED). Similar to XHED in other mammalian species, the primary phenotypic alterations include partially missing hair and a complete absence of the undercoat. Furthermore, the affected cat lacked most teeth, and the remaining teeth had an abnormal conical shape. The genetic investigation identified *EDA*:c.1042G>A, a single-nucleotide variant affecting a CpG dinucleotide and resulting in the p.Ala348Thr missense change as the most likely causative defect. A homologous missense variant, p.Ala349Thr, caused by recurring mutations of the homologous CpG dinucleotide has been reported in several human patients with XHED. To the best of our knowledge, we report the first instance of an *EDA*-related hypohidrotic ectodermal dysplasia in a cat.

## Figures and Tables

**Table 1:** Variants detected by whole-genome sequencing of the affected cat.

Filtering Step	Heterozygous Variants	Homozygous Variants ^1^
All variants	8,698,639	4,662,163
Private variants	101,930	3933
Private protein-changing variants	536	20
Private protein-changing variants in three candidate genes	0	1

^1^ Includes hemizygous variants on the X chromosome.

## Data Availability

The original genome sequence data presented in the study are openly available in European Nucleotide Archive at https://www.ebi.ac.uk/ena/browser/home, accessed on 18 April 2024. All other original contributions presented in the study are included in the article/Supplementary Materials, further inquiries can be directed to the corresponding author.

## Supplementary Materials

The following supporting information can be downloaded at: https://www.mdpi.com/article/10.3390/genes15070854/s1, Table S1: Accession numbers of 97 cat genome sequences; Table S2: Hematology results of the affected cat; Table S3: Serum biochemistry of the affected cat; Table S4: Urinalysis of the affected cat; Table S5: Private protein-changing private variants in the affected cat.
